# ^68^Ga-DOTATATE and ^68^Ga-Pentixafor PET/CT in a Patient with Castleman Disease of the Retroperitoneum

**DOI:** 10.3390/diagnostics14040372

**Published:** 2024-02-08

**Authors:** Rui Zuo, Lu Xu, Hua Pang

**Affiliations:** Department of Nuclear Medicine, The First Affiliated Hospital of Chongqing Medical University, No. 1 Youyi Road, Chongqing 400016, China; 2019110327@stu.cqmu.edu.cn (R.Z.); lunaxuxu@foxmail.com (L.X.)

**Keywords:** ^68^Ga-DOTATATE, ^68^Ga-pentixafor, PET/CT, Castleman disease

## Abstract

This is a case of a 42-year-old man with recurrent symptoms of dizziness and a newly found retroperitoneal mass with no ^131^I-MIBG uptake who was referred for restaging with ^68^Ga-DOTATATE PET/CT and local ^68^Ga-pentixafor PET/CT. The examinations both showed intense radioactivity uptake in the retroperitoneal mass and no abnormal uptake in the right adrenal nodule. Two lesions showed distinct properties of radioactivity uptake, which suggested the possibility of different sources. A postoperative pathological test revealed that the morphology and immunohistochemistry of the retroperitoneal mass was found to be consistent with Castleman disease, and the right adrenal gland was normal tissue.

**Figure 1:**
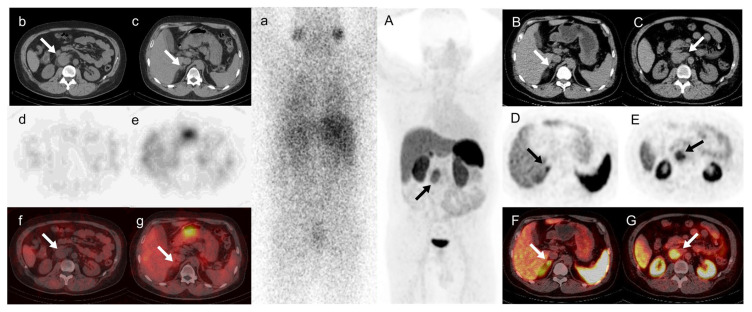
A 42-year-old man presented who had experienced repeated dizzy spells for 4 years. His blood pressure was 220/140 mmHg at the time of the attack. He complained of recent edema in both ankles and foamy urine. An upper abdominal CT scan revealed a suspicious retroperitoneal mass and a nodule on the lateral branch of the right adrenal gland. His catecholamine levels were slightly elevated, his aldosterone-to-renin ratio was 51.4 [pg/mL]/[uIU/mL], and captopril and saline inhibition tests were positive. Clinicians made a comprehensive diagnosis of primary aldosteronism, which may be associated with paraganglioma. Therefore, it is suggested to improve the relevant imaging examination, especially radionuclide functional imaging. ^131^I-MIBG SPECT images (**a**–**g**) indicated no radioactive uptake by the retroperitoneal mass or adrenal nodule. ^68^Ga-DOTATATE PET/CT images ((**A**) MIP; (**B**,**D**,**F**) arrows, adrenal nodule; (**C**,**E**,**G**) arrows, retroperitoneal mass) showed no significant increase in radioactive uptake in the right adrenal nodule. The SUVmax was 7.6 (the SUVmax of the left normal adrenal tissue was 7.2), while there was somatostatin receptor expression on the retroperitoneal mass with an SUVmax of 8.1. The SUVmax in the retroperitoneal mass is higher than in the right adrenal nodule, implying the mass had a higher level of CXCR4 expression. Castleman disease (CD) is a heterogeneous lymphoproliferative disease [1]. The key to its treatment lies in the evaluation of the systemic condition, but because of its varying locations and clinical manifestations, diagnosis can be quite challenging [2,3,4,5]. Thus, effective diagnostic methods are needed [6]. In this case, we found high uptake for both ^68^Ga-DOTATATE PET/CT and local ^68^Ga-pentixafor PET/CT. As we observed in this case, CD lesions exhibited notable ^68^Ga-DOTATATE absorption, which was also recently reported in two additional CD patients [4,7]. A CT scan of the former patient revealed a mediastinal mass after a neuroendocrine tumorectomy [4], and a scan of the latter patient revealed an inadvertent pancreatic mass [7]. To rule out a neuroendocrine condition, they both received ^68^Ga-DOTATATE PET/CT in a clinical setting. A surgical pathology test verified that they both had CD. Although the exact role of ^68^Ga-DOTATATE PET/CT in CD remains unclear, it interestingly presents a unique idea for the diagnosis of the disease: ^68^Ga-DOTATATE PET could be a helpful diagnostic tool. In this case, the high ^68^Ga-pentixafor uptake of CD was accidentally found in the process of evaluating primary aldosteronism with ^68^Ga-pentixafor PET/CT. This indicated that there is a certain degree of CXCR4 expression in CD, due to which CXCR4 may be highly expressed in a variety of solid tumors, and this receptor may be a suitable target for molecular imaging [8,9]. Of course, this also can assist in the clinical diagnosis of CD and reflect the expression of CXCR4. The focus of CD in this patient was in the retroperitoneum rather than the mediastinum, which is the most common location for unicentric CD [10]. Both ^68^Ga-DOTATATE and ^68^Ga-pentixafor PET/CT exhibited positive uptake, which has not previously been reported in a case of CD. We provide new imaging data for diagnosing CD. The positive uptake of both ^68^Ga-DOTATATE and ^68^Ga-pentixafor in lesions may further confirm this suspicion when the possibility of CD is considered clinically.

**Figure 2:**
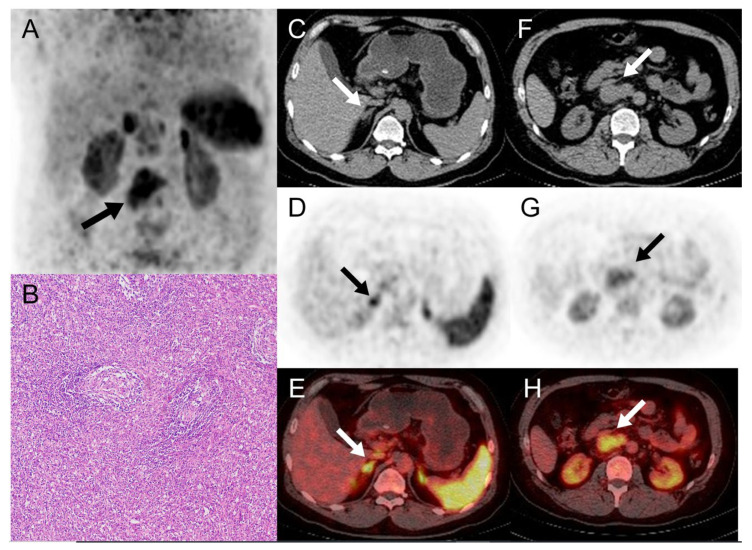
The MIP, axial CT, PET, and fusion PET/CT images of local ^68^Ga-pentixafor PET/CT showed no abnormal uptake in the right adrenal nodule ((**C**–**E**), arrows, SUVmax, 7.3; the contralateral normal adrenal gland, SUVmax, 7.1) and the presence of CXCR4 expression in the retroperitoneal mass (**A**,**F**–**H**), arrows, SUVmax, 6.2). Robot-assisted laparoscopic resection of the retrovena cava tumor and right adrenal gland was performed. A postoperative pathological test of the retroperitoneal mass revealed atypical large-cell lymphoid proliferation in a microscopic section of hematoxylin–eosin stain (**B**) (original magnification × 10), the immunohistochemistry analyses was consistent with Castleman’s disease ((**C**,**D**) hyaline vascular type), and the right adrenal nodule was normal tissue.

## Data Availability

Most data generated during this study are included in this published article. The data are available from the corresponding author on reasonable request, if you are confused about some of the data.
